# PGC-1α Regulates Exercise Intensity-Dependent Atrial Remodeling and Fibrillation in Rats

**DOI:** 10.14336/AD.2025.10621

**Published:** 2026-09-02

**Authors:** Jingwen Xiao, Jiancheng Zhang, Yan Zhang, Yu Jiang, Chenqi Yang, Xiaona Lin, Zhengnan Lin

**Affiliations:** ^1^The Department of cardiovascular medicine, Fuzhou First Hospital affiliated with Fujian Medical University, Fuzhou, Fujian, China.; ^2^The Department of cardiovascular medicine, Provincial Clinical College affiliated to Fujian Medical University, Fuzhou, Fujian, China.; ^3^Department of Pharmacology and Neuroscience, University of North Texas Health Science Center, Fort Worth, TX 76107, USA

**Keywords:** Atrial Fibrillation, PGC-1α, Exercise Intensity, Cardiac Remodeling, Metabolic Shift, Atrial Fibrosis

## Abstract

Exercise has well-documented cardiovascular benefits, but excessive training has been associated with an increased risk of atrial fibrillation (AF). The molecular mechanisms linking exercise intensity to atrial remodeling and AF susceptibility remain incompletely understood. Here, we investigated the effects of varying treadmill exercise intensities on atrial structure, metabolism, and electrophysiology in rats. AF inducibility was assessed using burst pacing, and atrial dimensions were evaluated by echocardiography. Histology was performed to quantify fibrosis and lipid accumulation. Metabolic and signaling pathways were examined through biochemical assays and Western blotting. We found that exercise intensity exhibited a nonlinear, J-shaped relationship with AF susceptibility. Moderate training (B-Mod) resulted in the lowest AF incidence and duration, whereas high-intensity training (B-Int) produced frequent and sustained episodes. Echocardiography revealed atrial enlargement in sedentary (B-Sed) and B-Int groups but preserved dimensions in B-Mod. Histological analysis showed marked fibrosis in B-Int but only minimal changes in B-Mod, along with progressive lipid deposition and impaired glucose handling at higher intensities. Importantly, PGC-1α expression paralleled AF susceptibility, peaking at moderate intensity, and was associated with decreased TGF-β and enhanced MAPK signaling. Pharmacological inhibition of PGC-1α with SR-18292 abolished these protective adaptations, increased fibrosis, disrupted glucose-lipid balance, and eliminated the correlation between Kv1.5 expression and AF resistance. In summary, moderate-intensity exercise protects against AF by optimizing atrial remodeling, metabolism, and electrophysiology through PGC-1α-dependent pathways. Both insufficient and excessive training impair these adaptations, increasing AF susceptibility. These findings identify PGC-1α as a central regulator of atrial health and a potential therapeutic target for AF prevention.

## INTRODUCTION

Atrial fibrillation (AF) is the most prevalent sustained cardiac arrhythmia and is strongly associated with increased risk of stroke, heart failure, cognitive impairment, and mortality [1]. Clinically, higher AF incidence is often observed in older individuals with a history of intensive physical activities or heavy labor, suggesting a complex relationship between long-term exercise habits and AF risk [2]. Epidemiological and experimental studies indicate a nonlinear, possibly U- or J-shaped, relationship between exercise intensity and AF incidence [3, 4]. Moderate physical activity appears protective, whereas both sedentary lifestyles [5, 6] and prolonged high-intensity endurance exercise [7, 8] are linked to greater AF risk. Proposed mechanisms include myocardial fibrosis, atrial remodeling, autonomic imbalance, and electrophysiological changes [9, 10], though the underlying molecular pathways remain poorly defined.

Peroxisome proliferator-activated receptor gamma coactivator-1 alpha (PGC-1α) is a key regulator of myocardial metabolism, controlling energy substrate utilization, mitochondrial biogenesis, and oxidative stress responses during exercise [11]. Under physiological conditions, PGC-1α supports efficient energy metabolism and structural stability, but both insufficient and excessive activation can drive metabolic disturbances, remodeling, and arrhythmogenesis [12, 13]. Reduced PGC-1α expression has been reported in AF patients, while interventions that enhance its activity can lower AF incidence [14]. However, whether PGC-1α directly contributes to AF development in the context of exercise remains unclear.

In this study, we investigated how exercise intensity modulates AF susceptibility and examined the role of PGC-1α in exercise-induced atrial remodeling and metabolic adaptation. We further evaluated whether reduced PGC-1α activation increases vulnerability to exercise-induced AF. Our findings provide mechanistic insights that may help guide personalized exercise prescriptions for AF prevention.

## MATERIALS AND METHODS

### Ethical Approval and Animal Housing

This study was conducted in accordance with the *Guide for the Care and Use of Laboratory Animals*, approved by the Animal Ethics Committee of Fujian Medical University, and complied with ARRIVE guidelines (Kilkenny et al., 2010; McGrath and Lilley, 2015). A total of 80 male Sprague-Dawley rats (10 weeks old; 250 ± 20 g; SPF Biotechnology Co., Ltd., Beijing) were housed in pairs under controlled conditions (22 ± 2°C; 40-70% humidity; 12-hour light/dark cycle) with free access to food and water.

### Grouping and Experimental Procedures

Rats were randomly assigned to two main groups: blank and PGC-1α inhibitor. Each group was further divided into four subgroups according to exercise intensity: sedentary (Sed), low-intensity (Low, 12 m/min), moderate-intensity (Mod, 16 m/min), and intermittent high-intensity (Int, 22 m/min), yielding eight experimental groups: B-Sed, B-Low, B-Mod, B-Int, SR-Sed, SR-Low, SR-Mod, and SR-Int.

After a one-week acclimation, rats in the inhibitor groups received intraperitoneal injections of SR-18292 (45 mg/kg, twice weekly). Two weeks later, all animals began an 8-week treadmill protocol while continuing SR-18292 treatment. The protocol included a one-week adaptation phase followed by structured training at the designated intensities for 30 minutes per day, while sedentary rats remained inactive.

### Echocardiographic Measurements

Rats were anesthetized with pentobarbital sodium (40 mg/kg, i.p.) and subjected to transthoracic echo-cardiography (11.5 MHz transducer; depth, 3.5 cm; frame rate, 90 fps). Left atrial diameter (LAD) and area (LAA) were measured from parasternal long-axis and apical four-chamber views prior to mitral valve opening [15].

### Electrophysiological Assessments

Anesthesia was maintained with pentobarbital sodium (40 mg/kg, i.p.). Surface ECG and esophageal electrode catheter recordings were used for programmed stimulation to assess the sinoatrial interval (SN), atrioventricular node refractory period (AVNRP), atrial refractory period (ARP), and sinoatrial node recovery time (SNRT). Rapid atrial pacing (30 Hz; 2-s pacing, 10-s rest; repeated twice with 5 cycles) was performed to induce AF, defined as an irregular atrial rhythm lasting >1 s or a ventricular rate ≥800 bpm. Persistent AF was defined as an episode lasting ≥30 s.

### Histological Analysis

After measurements, atrial tissues were fixed in 4% paraformaldehyde (PFA), embedded in paraffin, and sectioned (4-6 µm) for fibrosis assessment using Masson’s trichrome staining. For lipid deposition, tissues were embedded in OCT, frozen, sectioned (8-10 µm), and stained with Oil Red O. Microscopic images were acquired (OCUS-MGU-00003, Grundium, Finland) and analyzed with Image-Pro Plus 6.0, with at least 100 randomly selected myocytes evaluated per sample.

### Myocardial FFA and Glucose Measurements

Atrial tissues stored at -80 °C were analyzed for free fatty acid (FFA) and glucose levels using commercial kits (FFA: ab65341; glucose: ab65333; Abcam), with quantification based on fluorescence absorbance.

### Western Blotting

Proteins from atrial tissues were extracted using PMSF-containing lysis buffer, quantified by BCA assay, separated by SDS-PAGE (5-10%), and transferred onto PVDF membranes. After blocking with 5% BSA for 2 h at room temperature, membranes were incubated overnight at 4 °C with primary antibodies against GAPDH (1:100,000; 60004-1-Ig, Proteintech), PGC-1α (1:10,000; 66369-1-Ig, Proteintech), p38 MAPK (1:5,000; 14064-1-AP, Proteintech), TGF-β1 (1:2,000; 21898-1-AP, Proteintech), and KCNA5 (1:1,500; 21659-1-AP, Proteintech). Membranes were then incubated with HRP-conjugated secondary antibodies (1:5,000; 2 h, room temperature), followed by visualization using enhanced chemiluminescence (ECL; Beyotime, China) and densitometric analysis with ImageJ.

### Statistical Analysis

Data are presented as mean ± SD. Normality was assessed using the Shapiro-Wilk test. Normally distributed data were analyzed with Student’s *t*-test (two groups) or one-way ANOVA followed by Tukey’s post hoc test (more than two groups). Non-normally distributed data were analyzed with the Mann-Whitney test (two groups) or the Kruskal-Wallis test followed by Dunn’s post hoc test (more than two groups). Statistical significance was set at *p* < 0.05.

**Figure 1.**
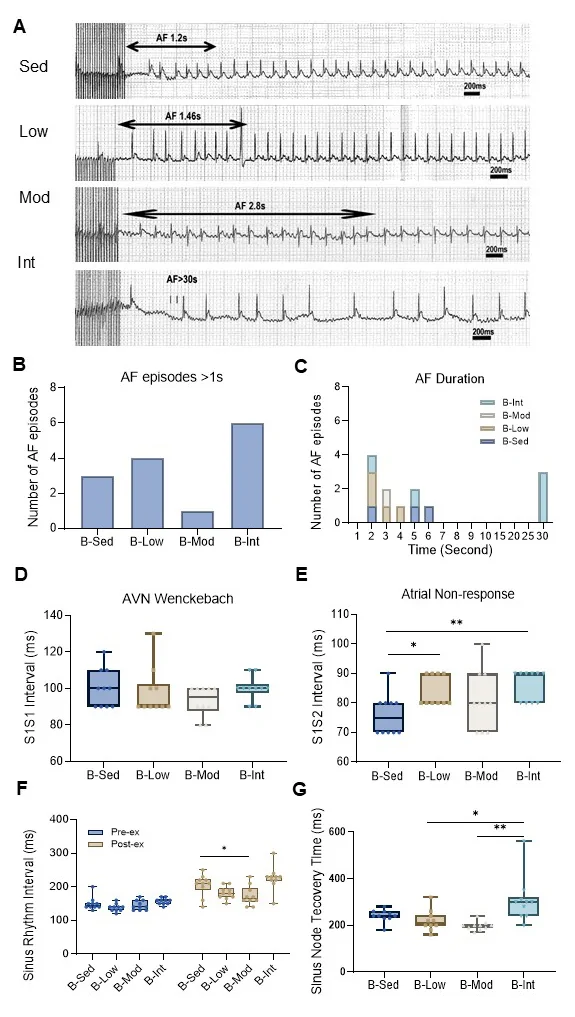
**Exercise intensity modulates atrial fibrillation (AF) susceptibility and electrophysiological parameters**. (**A**) Representative intracardiac electrograms showing AF induction via 30-Hz, 10-V burst pacing for 2 seconds in sedentary (B-Sed), low-intensity (B-Low), moderate-intensity (B-Mod), and high-intensity (B-Int) exercise groups. AF episode durations are indicated above each trace. (**B**) Number of AF episodes (>1 s) per group: B-Sed = 3; B-Low = 4; B-Mod = 1; B-Int = 6. (**C**) Distribution of AF episode durations; maximum durations: B-Sed = 5.8 s, B-Low = 3.6 s, B-Mod = 1.46 s, B-Int ≥ 30 s. (**D**) Wenckebach cycle length (WCL) assessed by S1S1 pacing protocol. (**E**) Atrial refractory period (ARP) measured using S1S2 pacing. (**F**) Sinus rhythm (SR) intervals measured before (Pre) and after the exercise protocol. (**G**) Sinus node recovery time (SNRT) following 30-second pacing. Data in panels D, E, and G were analyzed using the Mann-Whitney U test (two-group comparisons) or Kruskal-Wallis test with Dunn’s post hoc test (multiple comparisons); data in panel F were analyzed using one-way ANOVA followed by Tukey’s post hoc test. All groups: *n* = 10 rats. *P* < 0.05; *P* < 0.01.

**Figure 2.**
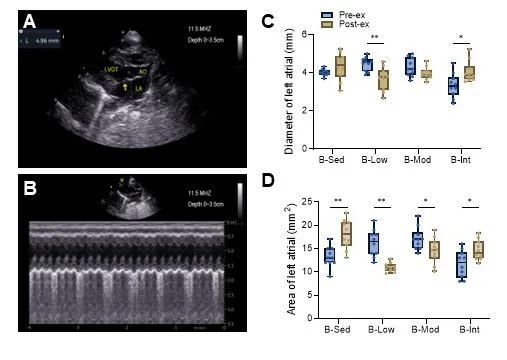
**Effects of exercise intensity on left atrial (LA) remodeling assessed by transthoracic echocardiography**. (**A**) Parasternal long-axis view showing measurement of the LA anteroposterior diameter prior to mitral valve opening (arrow). (**B**) M-mode echocardiogram from the same view confirming preserved left ventricular systolic function. (**C**) LA diameter and (D) LA area index measured before (Pre) and after the 8-week exercise protocol in sedentary (B-Sed), low-intensity (B-Low), moderate-intensity (B-Mod), and high-intensity (B-Int) groups. All data were normally distributed and analyzed using Student’s *t*-test. Sample size: *n* = 10 rats per group. *P* < 0.05; *P* < 0.01.

## RESULTS

### Exercise Intensity Differentially Modulates Atrial Fibrillation Susceptibility

To investigate the effects of exercise intensity on AF susceptibility, rats underwent a graded treadmill training protocol. The heart weight-to-body weight ratio (HW/BW) increased progressively with exercise intensity (Table 1). Representative intracardiac electrograms illustrating AF induction by burst pacing are shown in Figure 1A. AF inducibility followed a J-shaped relationship with exercise intensity: the B-Int group exhibited the highest number of episodes (6/10), whereas B-Mod had the lowest (1/10) (Fig. 1B, Table 1). Likewise, the longest AF durations were observed in B-Int (≥30 s), with the shortest in B-Mod (1.46 s) (Fig. 1C, Table 1). Additional arrhythmic events, including premature ventricular contractions (PVCs) and atrioventricular (AV) block, were most frequent in B-Sed and B-Int groups.

**Table 1:** Electrophysiological characteristics across exercise intensities.

Group	HW/BW (g/kg)	AF Episodes (n)	Max AF Duration (s)	PVCs (n)	AV Block (n)
**B-Sed**	3.16 ± 0.56	3	5.8	2	0
**B-Low**	3.26 ± 0.33	4	3.6	1	0
**B-Mod**	3.28 ± 0.34	1	1.46	0	0
**B-Int**	3.42 ± 0.27	6	≥30	0	3

Heart weight-to-body weight ratio (HW/BW), atrial fibrillation (AF) inducibility, maximum AF episode duration, premature ventricular contractions (PVCs), and atrioventricular (AV) block events recorded in each group (n = 10/group). Data are presented as mean ± SD.

### Exercise-Induced Structural Remodeling of the Left Atrium

**Figure 3.**
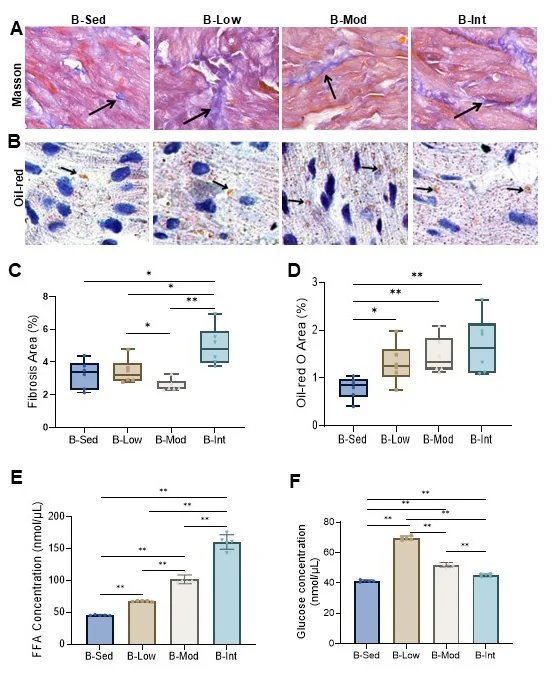
**Effects of exercise intensity on atrial fibrosis, lipid accumulation, and metabolic substrate levels**. (**A**) Representative Masson’s trichrome-stained sections (×40) showing interstitial collagen (blue) and myofibers (red); arrows indicate fibrotic regions. (**B**) Oil Red O-stained sections (×80) highlighting lipid droplets (orange-red), nuclei (blue), and phospholipids (pink); arrows indicate lipid deposits. (**C**) Quantification of fibrotic area expressed as a percentage of total atrial tissue. (**D**) Quantification of Oil Red O-positive area. (**E**) Free fatty acid (FFA) concentrations and (F) glucose concentrations in atrial tissue homogenates. Data in panels C and E were analyzed using the Mann-Whitney U test or Kruskal-Wallis test with Dunn’s post hoc analysis. Data in panels D and F were analyzed using Student’s *t*-test or one-way ANOVA with Tukey’s post hoc test. Sample sizes: *n* = 6 (C, D); *n* = 5 (E, F). *P* < 0.05; *P* < 0.01.

Echocardiographic assessment revealed significant left atrial dilation in B-Sed and B-Int groups, as indicated by increased diameter and area measurements, while B-Mod preserved near-normal atrial dimensions (Fig. 2C, D). Measurement landmarks are demonstrated in the parasternal long-axis view (Fig. 2A), and M-mode confirmed preserved left ventricular systolic function (Fig. 2B). Histological analysis corroborated these findings: Masson’s trichrome staining revealed marked fibrosis in B-Int, moderate fibrosis in B-Low, and minimal fibrosis in B-Mod (Fig. 3A, C), supporting a protective structural remodeling profile in the moderate-intensity group.

### Exercise Intensity Alters Atrial Lipid Accumulation and Substrate Utilization

Oil Red O staining demonstrated a clear intensity-dependent increase in lipid accumulation within atrial tissue (Fig. 3B, D). Free fatty acid (FFA) levels increased progressively with exercise intensity, while glucose content declined (Fig. 3E, F). Notably, B-Mod exhibited an intermediate lipid profile with significantly elevated glucose levels, indicative of enhanced metabolic flexibility. In contrast, B-Int demonstrated the highest lipid burden and lowest glucose availability, consistent with metabolic stress and potential lipotoxic remodeling.

**Figure 4.**
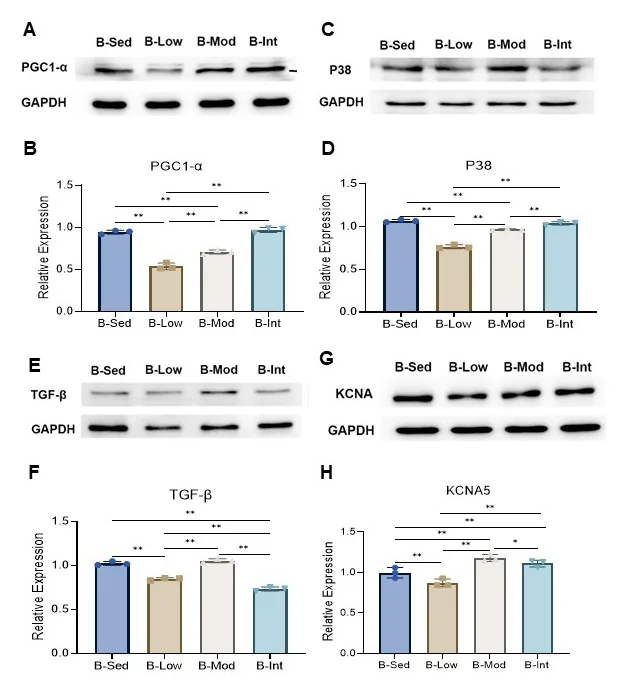
**Protein expression in atrial tissue across exercise intensities**. (**A**) Representative Western blot showing PGC-1α expression in atrial tissue from rats subjected to varying exercise intensities. GAPDH was used as a loading control. (**B**) Quantification of PGC-1α protein levels normalized to GAPDH. (C, D) Expression of p38 MAPK and its corresponding quantification. (E, F) Expression of TGF-β and its corresponding quantification. (G, H) Expression of Kv1.5 (KCNA5) and corresponding quantification. Data in panels B, D, and F were analyzed using one-way ANOVA followed by Tukey’s post hoc test. Data in panel H were analyzed using the Mann-Whitney U test or Kruskal-Wallis test with Dunn’s post hoc test. Sample size: *n* = 3 per group. *P* < 0.05; *P* < 0.01.

### PGC-1α Expression Reflects Exercise Intensity and Modulates Atrial Remodeling Pathways

Western blot analysis revealed that PGC-1α expression increased with exercise intensity, peaking in B-Int and remaining moderately elevated in B-Mod (Fig. 4A, B). Although B-Int exhibited the highest absolute levels, the functional adaptations, such as reduced fibrosis and improved metabolism, were most pronounced in B-Mod, suggesting that excessive PGC-1α activation may be maladaptive. The upstream regulator p38 MAPK expression increased linearly across intensities (Fig. 4C, D), while downstream profibrotic marker TGF-β expression was lowest in B-Mod and significantly elevated in B-Int (Fig. 4E, F). Kv1.5, a potassium channel involved in atrial repolarization, was upregulated in all exercise-trained groups (Fig. 4G, H), potentially contributing to electrical stability.

**Table 2:** Electrophysiological parameters following PGC-1α inhibition.

Group	AF Episodes (n)	Max AF Duration (s)	PVCs (n)	AV Block (n)
**SR-Sed**	0	0	1	0
**SR-Low**	3	2.1	0	0
**SR-Mod**	10	5.12	2	1
**SR-Int**	8	4.2	0	2

Atrial fibrillation (AF) inducibility, maximum AF episode duration, premature ventricular contractions (PVCs), and atrioventricular (AV) block events in PGC-1α inhibitor-treated groups (n = 10/group). SR = inhibitor-treated groups at corresponding exercise intensities.

### PGC-1α Inhibition Disrupts Exercise-Induced Atrial Adaptations

**Figure 5.**
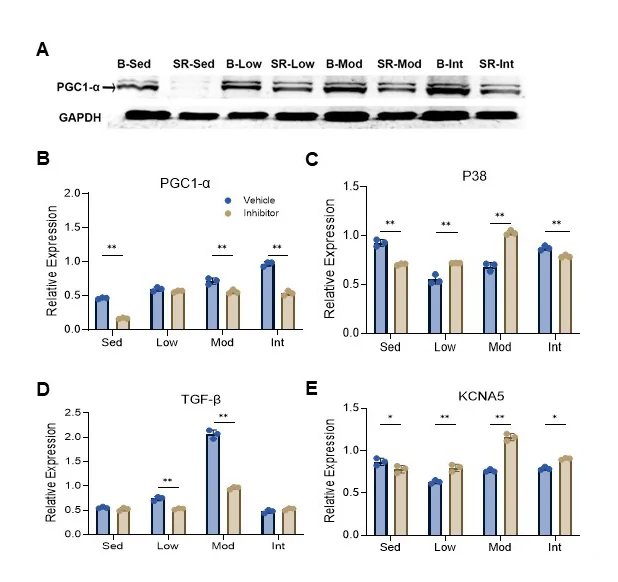
**Effect of PGC-1α inhibition on expression of key atrial proteins across exercise intensities**. (**A**) Western blot analysis of PGC-1α expression in atrial tissue from rats subjected to varying exercise intensities, with or without PGC-1α inhibition (SR groups). GAPDH was used as a loading control. (**B**) Quantification of PGC-1α expression. (**C**) Relative expression of p38 MAPK. (**D**) Relative expression of TGF-β. (**E**) Relative expression of Kv1.5 (KCNA5). Data in panels C and E were normally distributed and analyzed using Student’s *t*-test. Data in panels B and D were analyzed using the Mann-Whitney U test. Sample size: *n* = 3 per group. *P* < 0.05; *P* < 0.01.

**Figure 6.**
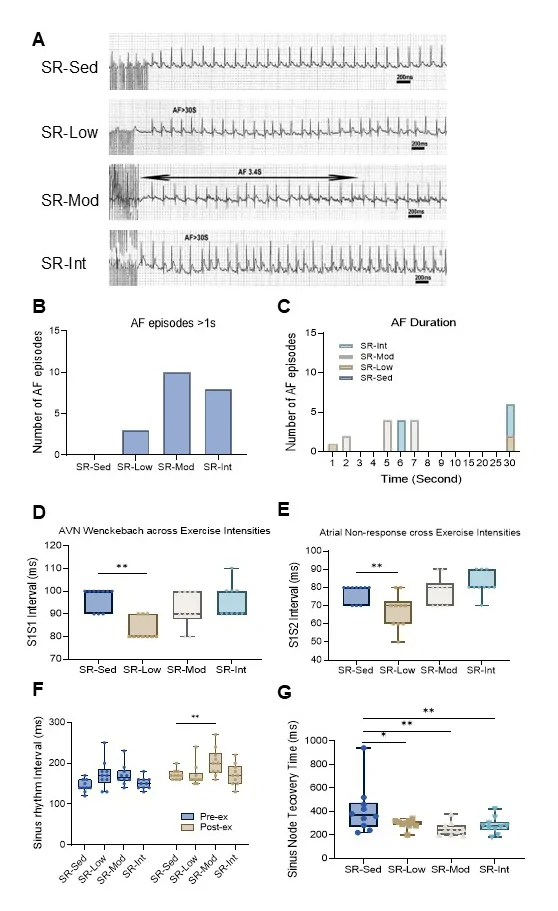
**Electrophysiological effects of PGC-1α inhibition across exercise intensities**. (**A**) Representative intracardiac electrograms showing AF induction via burst pacing in sedentary (SR-Sed), low-intensity (SR-Low), moderate-intensity (SR-Mod), and high-intensity (SR-Int) exercise groups treated with the PGC-1α inhibitor. AF episode durations are indicated on each trace. (**B**) Number of AF episodes >1 second per group. (**C**) Distribution of AF episode durations across groups. (**D**) Wenckebach cycle length (S1S1 interval) measured during programmed stimulation. (**E**) Atrial non-response assessed using S1S2 pacing protocol. (**F**) Sinus rhythm intervals before and after exercise. (**G**) Sinus node recovery time (SNRT) following 30-second pacing. Data in panels D-G were analyzed using the Mann-Whitney U test (two-group comparisons) or Kruskal-Wallis test with Dunn’s post hoc test (multiple comparisons). Sample size: *n* = 10 rats per group. *P* < 0.05; P < 0.01.

In the PGC-1α inhibitor (SR-18292)-treated groups, AF susceptibility increased markedly in SR-Mod (10 episodes) and SR-Int (8 episodes), with prolonged durations of up to 5.12 s and 4.2 s, respectively (Fig. 6B, C; Table 2). Representative electrograms are provided in Figure 6A. The SR-Mod group showed the highest arrhythmic burden, including AV block and multiple PVCs (Table 2). Inhibition of PGC-1α abolished the intensity-dependent expression patterns observed in non-inhibited groups and disrupted MAPK/TGF-β signaling (Fig. 5A-D). TGF-β expression was notably elevated in SR-Mod, aligning with the observed increase in fibrosis.

PGC-1α inhibition also impaired atrial conduction and pacemaker function. The SR-Mod group demonstrated prolonged sinus node recovery time (SNRT), atrial refractory period (ARP), and Wenckebach cycle length (WCL) compared to vehicle-treated controls (Fig. 6D-G), indicating compromised electrophysiological integrity.

### Structural and Metabolic Consequences of PGC-1α Suppression

**Figure 7.**
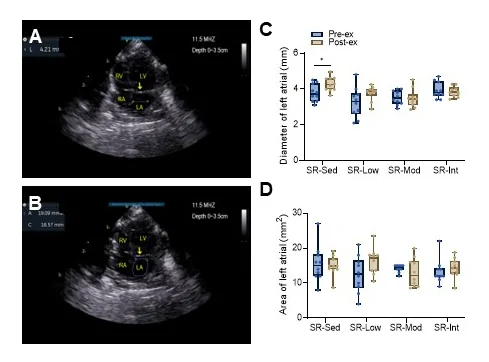
**Assessment of left atrial remodeling in PGC-1α inhibitor-treated rats using transthoracic echocardiography**. (**A**) Apical four-chamber view showing measurement of left atrial (LA) mediolateral diameter before mitral valve opening. (**B**) Apical four-chamber view showing measurement of LA volume index before mitral valve opening. (**C**) LA diameter and (D) LA area index measured before (Pre) and after exercise in SR-treated groups (SR-Sed, SR-Low, SR-Mod, SR-Int). All data were normally distributed and analyzed using Student’s *t*-test. Sample size: *n* = 10 rats per group. *P* < 0.05.

**Figure 8.**
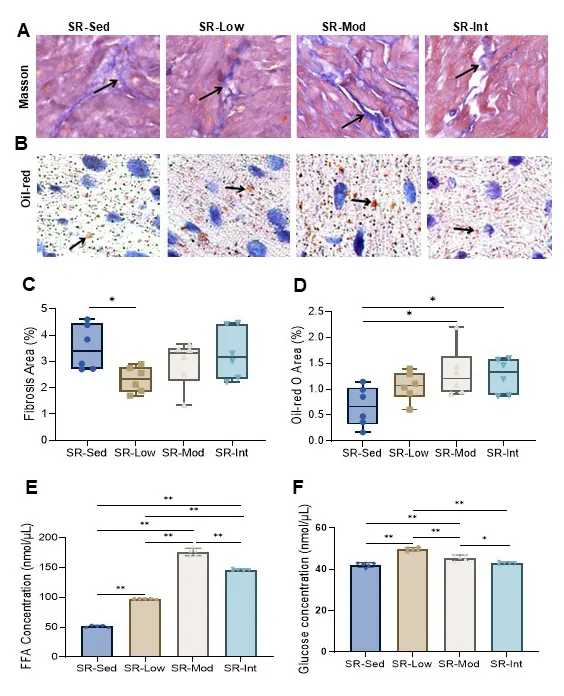
**Effects of PGC-1α inhibition on atrial fibrosis, lipid accumulation, and metabolic substrates**. (**A**) Representative Masson’s trichrome-stained sections highlighting interstitial collagen (blue) and myofibers (red); arrows indicate fibrotic regions. (**B**) Oil Red O-stained sections showing lipid deposits (orange-red), nuclei (blue), and phospholipids (pink); arrows indicate lipid accumulation. (**C**) Quantification of fibrotic area as a percentage of total atrial tissue. (**D**) Quantification of Oil Red O-positive area. (**E**) Free fatty acid (FFA) concentrations and (F) glucose concentrations in atrial tissue homogenates. All data were normally distributed and analyzed using one-way ANOVA with Tukey’s post hoc test. Sample size: *n* = 6 (C, D); *n* = 5 (E, F). *P* < 0.05; *P* < 0.01.

Echocardiographic and histological analyses revealed significant left atrial enlargement and collagen deposition in SR-Low and SR-Mod groups (Fig. 7C, D; Fig. 8A, C). The SR-Mod group exhibited the most severe fibrotic remodeling. Oil Red O staining and biochemical assays showed substantial lipid accumulation and elevated FFA levels in SR-Mod (Fig. 8B, D, E), along with markedly reduced glucose levels (Fig. 8F). These findings suggest a shift toward lipotoxicity and impaired substrate flexibility under PGC-1α suppression.

Despite preserved Kv1.5 expression in the SR exercise groups, the previously observed association between Kv1.5 expression and reduced AF susceptibility was lost (Fig. 5E). This implies that PGC-1α-mediated regulation of electrophysiological properties may involve additional factors beyond channel expression alone.

**Figure 9.**
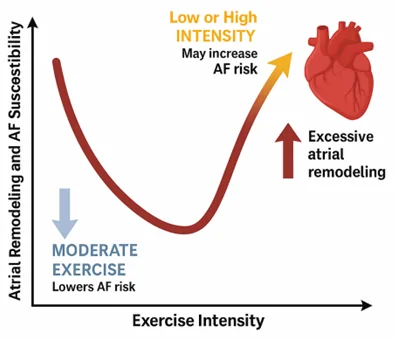
**Schematic Illustration of Exercise Intensity-Dependent Effects on Atrial Remodeling and AF Susceptibility**. A conceptual J-shaped curve depicts the relationship between exercise intensity and atrial health. Moderate-intensity exercise is associated with balanced upregulation of PGC-1α, promoting adaptive atrial remodeling, improved metabolic function, and reduced susceptibility to AF. In contrast, both low- and high-intensity exercise are associated with inadequate or excessive PGC-1α activity, respectively, contributing to structural remodeling, lipid deposition, fibrosis, and increased AF risk. This model emphasizes the role of exercise intensity in modulating atrial substrate vulnerability through PGC-1α-mediated pathways.

Collectively, the findings support a J-shaped relationship between exercise intensity and AF risk, mediated by PGC-1α-dependent regulation of structural, metabolic, and electrophysiological remodeling. This relationship is depicted schematically in Figure 9, highlighting the central role of PGC-1α in promoting adaptive remodeling at moderate exercise intensity, and maladaptive changes when PGC-1α activation is either insufficient or excessive.

## DISCUSSION

This study demonstrates a nonlinear, J-shaped relationship between exercise intensity and atrial fibrillation (AF) susceptibility in a rat model. Moderate-intensity training was associated with the lowest AF inducibility and duration, while both sedentary behavior and high-intensity exercise increased arrhythmic vulnerability. These findings reflect clinical observations that moderate physical activity protects against AF, whereas both inactivity and excessive endurance training may elevate risk.

The protective effects of moderate exercise extended to atrial structure, metabolism, and electrophysiology. B-Mod animals exhibited preserved atrial size, minimal fibrosis, and enhanced glucose utilization. In contrast, B-Int and B-Sed groups showed atrial dilation, interstitial fibrosis, lipid accumulation, and electrophysiological instability. These results suggest that appropriate exercise intensity promotes adaptive remodeling, while deviation in either direction contributes to substrate vulnerability.

A central finding of this study is the pivotal role of PGC-1α in mediating exercise-induced atrial adaptations. PGC-1α expression followed a nonlinear trend—moderately elevated in B-Mod, markedly increased in B-Int, and low in B-Sed. Importantly, only moderate upregulation of PGC-1α was associated with protective effects, whereas excessive expression under high-intensity exercise coincided with profibrotic signaling and metabolic dysfunction. This suggests that PGC-1α operates within an optimal functional window, beyond which maladaptive remodeling may occur.

Supporting this interpretation, p38 MAPK, an upstream regulator of PGC-1α, increased with exercise intensity, while TGF-β, a downstream marker of fibrosis, was lowest in B-Mod but elevated in B-Int. Moderate activation of PGC-1α appears to restrain fibrotic remodeling, potentially via suppression of TGF-β/Smad signaling or through improved mitochondrial dynamics and oxidative balance, as suggested in previous studies [16-19].

Metabolic analysis further revealed intensity-dependent shifts in substrate use. Moderate exercise enhanced glucose uptake and limited lipid accumulation, consistent with efficient metabolic adaptation [21-25]. High-intensity training promoted excessive free fatty acid (FFA) accumulation and reduced glucose content, features indicative of lipotoxic stress [26-28]. These metabolic changes paralleled alterations in structural integrity and AF susceptibility.

Electrophysiologically, Kv1.5, a voltage-gated potassium channel involved in atrial repolarization, was upregulated in all exercise-trained groups. This may have contributed to the shortened atrial refractory period and reduced AF inducibility. However, this relationship was abolished in PGC-1α-inhibited animals, despite maintained Kv1.5 expression, suggesting that upstream metabolic and structural balance is necessary for the functional benefit of ion channel remodeling [32-36].

Pharmacological inhibition of PGC-1α with SR-18292 disrupted the beneficial effects of moderate exercise. SR-Mod animals showed increased AF episodes, atrial fibrosis, lipid deposition, and impaired conduction parameters. The abrogation of TGF-β suppression and the loss of metabolic flexibility reinforce the central role of PGC-1α in coordinating adaptive responses. Notably, the dissociation between Kv1.5 expression and AF resistance in these groups further emphasizes the requirement of an integrated remodeling program.

Taken together, these findings support a model in which PGC-1α acts as a molecular hub that integrates exercise signals to fine-tune atrial structure, metabolism, and electrical function. Moderate-intensity training optimally activates this network, whereas both insufficient and excessive activation can promote pathological remodeling. This mechanistic insight is summarized in the conceptual model (Fig. 9), which depicts how exercise intensity modulates AF risk via PGC-1α-mediated pathways.

### Clinical Implications

While this study was conducted in a rodent model, the results align with epidemiological data suggesting that moderate exercise reduces AF risk, while both low and high levels increase it [3, 4, 37-40]. The identification of PGC-1α as a mediator of this phenomenon raises the possibility that PGC-1α expression or downstream markers could serve as biomarkers for atrial health. Moreover, targeting PGC-1α or its regulatory pathways may offer new therapeutic strategies for AF prevention, particularly in populations with high cardiovascular stress or metabolic risk.

### Limitations and Future Directions

This study has several limitations. First, it employed a rat model, which differs from human AF in its substrate and progression. Second, only male animals were used, precluding analysis of sex-specific effects. Third, pharmacological inhibition of PGC-1α may have off-target effects. Finally, the long-term persistence of exercise-induced changes was not evaluated.

Future research should explore the dose-response relationship between exercise intensity and PGC-1α activity in both sexes and across age groups. Longitudinal studies are needed to determine whether maladaptive remodeling is reversible. Clinical trials assessing whether modulation of PGC-1α, or guided exercise prescriptions, can reduce AF incidence are also warranted.

### Conclusion

In conclusion, moderate-intensity exercise promotes adaptive atrial remodeling and protects against AF through PGC-1α-dependent pathways. Both insufficient and excessive training impair these adaptations, increasing AF susceptibility. PGC-1α emerges as a critical integrator of structural, metabolic, and electro-physiological responses to exercise, and represents a promising therapeutic target for AF prevention and management.
